# Regulation of cardiomyocyte adhesion and mechanosignalling through distinct nanoscale behaviour of integrin ligands mimicking healthy or fibrotic extracellular matrix

**DOI:** 10.1098/rstb.2022.0021

**Published:** 2022-11-21

**Authors:** William Hawkes, Emilie Marhuenda, Paul Reynolds, Caoimhe O'Neill, Pragati Pandey, Darren Graham Samuel Wilson, Mark Freeley, Da Huang, Junquiang Hu, Sasha Gondarenko, James Hone, Nikolaj Gadegaard, Matteo Palma, Thomas Iskratsch

**Affiliations:** ^1^ School of Engineering and Materials Science, Queen Mary University of London, London E1 4NS, UK; ^2^ Department of Chemistry, Queen Mary University of London, London E1 4NS, UK; ^3^ School of Engineering, University of Glasgow, Glasgow G12 8QQ, UK; ^4^ Department of Mechanical Engineering, Columbia University, New York, NY 10027, USA

**Keywords:** cardiomyocyte mechanosensing, extracellular matrix, nanopatterning, traction force, DNA origami, integrins

## Abstract

The stiffness of the cardiovascular environment changes during ageing and in disease and contributes to disease incidence and progression. Changing collagen expression and cross-linking regulate the rigidity of the cardiac extracellular matrix (ECM). Additionally, basal lamina glycoproteins, especially laminin and fibronectin regulate cardiomyocyte adhesion formation, mechanics and mechanosignalling. Laminin is abundant in the healthy heart, but fibronectin is increasingly expressed in the fibrotic heart. ECM receptors are co-regulated with the changing ECM. Owing to differences in integrin dynamics, clustering and downstream adhesion formation this is expected to ultimately influence cardiomyocyte mechanosignalling; however, details remain elusive. Here, we sought to investigate how different cardiomyocyte integrin/ligand combinations affect adhesion formation, traction forces and mechanosignalling, using a combination of uniformly coated surfaces with defined stiffness, polydimethylsiloxane nanopillars, micropatterning and specifically designed bionanoarrays for precise ligand presentation. Thereby we found that the adhesion nanoscale organization, signalling and traction force generation of neonatal rat cardiomyocytes (which express both laminin and fibronectin binding integrins) are strongly dependent on the integrin/ligand combination. Together our data indicate that the presence of fibronectin in combination with the enhanced stiffness in fibrotic areas will strongly impact on the cardiomyocyte behaviour and influence disease progression.

This article is part of the theme issue ‘The cardiomyocyte: new revelations on the interplay between architecture and function in growth, health, and disease’.

## 1. Introduction

The cardiac extracellular matrix (ECM) provides structural support for cardiomyocytes, the contractile cells of the heart. Additionally, the chemical and mechanical composition of the ECM is sensed by the cardiomyocytes and influences their differentiation, maturity and function. Importantly, the ECM's composition, structure and mechanics change during development and in heart disease. Changes include different collagen isoforms, collagen cross-linking, upregulation of fibronectin in the basal lamina and increasing myocardial stiffness from approximately 1 kPa in the fetal heart to approximately 10 kPa in the healthy adult heart and 50–150 kPa in the diseased, fibrotic myocardium [1]. These changes are sensed through different pathways, but especially through integrin adhesion and downstream mechanosignalling [2–5]. The transmembrane integrin receptors enable inside-out and outside-in mechanotransduction [6]. On the outside of the cell, they bind to ECM molecules, such as laminin, fibronectin or collagen, depending on the integrin isoforms. On the cytoplasmic face they connect to the actin cytoskeleton via an adhesion complex with talin at its core. Actomyosin forces on talin can open cryptic binding sites for vinculin (depending on the stiffness of the ECM), which reinforces the adhesion through providing further connections to the actin cytoskeleton [6,7].

Cardiomyocyte integrins are primarily located at specific circumferential adhesion sites, the so-called costameres, although in two-dimensional culture integrins are further found in focal adhesion-like structures [8]. The expression profile of the cardiomyocyte integrin is highly regulated, and specific expression patterns are activated during developmental, neonatal, healthy adult and diseased states [8–13]. Specifically, the laminin binding *α*7*β*1 integrin becomes the primary receptor in adulthood; however, the fibronectin binding *α*5*β*1 integrins are the predominant subtype during development and in infarcted, or ischaemic hearts, where increasing amounts of fibronectin are deposited by cardiac fibroblasts [1,13].

However, different integrins vary in their ligand-binding characteristics, dynamics and nanoscale organization [1,14–16] and therefore, not only ECM stiffness but also the specifics of the ECM ligand/integrin are expected to influence the adhesion mechanosignalling. Indeed, changes to adhesion composition have been linked to postnatal heart development as well as to a maladaptive response and progression to heart failure, but details remain unclear [1].

To address this, we sought here to investigate how different cardiomyocyte integrin/ligand combinations affect adhesion formation, traction forces and mechanosignalling, using a combination of: (i) uniformly coated surfaces with defined stiffness, (ii) polydimethylsiloxane (PDMS) nanopillars, (iii) micropatterning and (iv) specifically designed bionanoarrays for precise ligand presentation.

Using this combined approach, our results consistently demonstrate that neonatal rat cardiomyocytes (NRCs) on fibronectin have enhanced spreading compared to cells cultured on laminin, at physiological and fibrotic stiffness. Moreover, NRCs cultured on fibronectin exhibit greater traction forces than cells cultured on nanopillars coated with laminin. Additionally, micropatterning revealed greater vinculin enrichment to fibronectin adhesions, suggesting altered rigidity sensing and mechanotransduction. Finally, our use of DNA origami bionanoarrays identifies unique properties of both laminin- or fibronectin (RGD)-binding integrins, especially differences in minimal local and global ligand concentrations needed for stable adhesion formation and spreading. Together our results demonstrate that integrin–ligand pairing critically influences the mechanosignalling of cardiomyocytes.

## 2. Results

### (a) Cardiomyocyte morphology and spread area are affected by the extracellular matrix composition

Because of changing ECM composition and co-regulation of integrin isoforms we first wanted to establish differences in cardiomyocyte phenotype dependent on the ECM stiffness and composition. NRCs are a popular model for cardiomyocyte studies owing to their relative maturity (e.g. compared to standard induced pluripotent stem cell-derived cardiomyocytes (IPSC-CM) models) and ease of culture (e.g. compared to adult cardiomyocytes, which start to dedifferentiate immediately after plating [17]). Indeed, NRCs are well suited to study the interaction of cardiomyocytes with different ECM components since they express high levels of both laminin- and fibronectin-binding integrins (electronic supplementary material, figure S1) [12].

**Figure 1.**
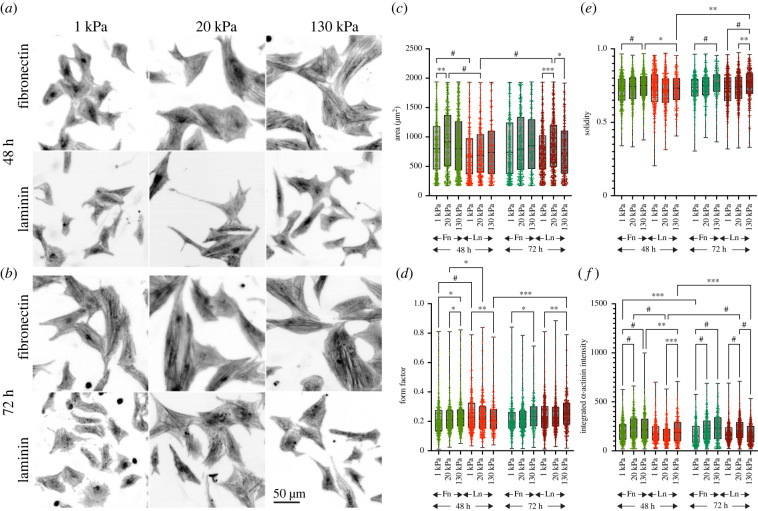
(*a*,*b*) NRCs on fibronectin- or laminin-coated PDMS substrates with defined stiffness (1 kPa, 20 kPa, 130 kPa) were fixed after 48 h (*a*) and 72 h (*b*) and stained for α-actinin. Cell morphology (*c–e*) and α-actinin intensity (*f*) were quantified from three independent experiments with more than 300 cells per condition. *N* = 3 independent experiments with more than 400 cells per condition. *p < 0.05, **p < 0.01, ***p < 0.001, ^#^*p* < 0.0001. *p-*values from one-way ANOVA (fibronectin versus laminin for each condition) and Tukey correction for multiple comparisons. Fn, fibronectin; Ln, laminin. Scale bar, 50 µm.

We first analysed cell area and shape of NRCs when plated on PDMS surfaces with different stiffness that were coated with either fibronectin or laminin (figure 1). On fibronectin, cells started showing a characteristic mechanosensitive behaviour after 48 h. In agreement with our previous findings, the largest cardiomyocyte areas and strongest α-actinin staining were found on fibronectin on 20 kPa surfaces [5], whereas cell shape changes were more pronounced on 130 kPa with an increased solidity and form factor, in agreement with a more spindle-shaped appearance (figure 1*d*,*e*). By contrast, cardiomyocytes on laminin did not show significant stiffness-dependent differences after 48 h. However, after 72 h cardiomyocytes also displayed a similar mechanosensitive response on laminin, with the largest areas and strongest actinin staining on 20 kPa and progressively increasing solidity and form factor with increasing stiffness (figure 1).

### (b) Cardiomyocyte adhesion forces are increased on fibronectin compared to laminin

**Figure 2.**
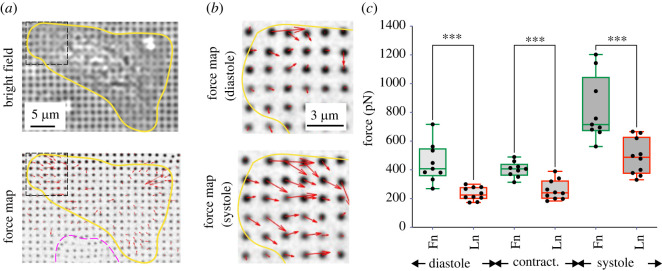
NRCs generate higher traction forces on fibronectin versus laminin. (*a–c*) NRC traction forces were analysed using PDMS nanopillars coated with quantum dots. (*a*) Bright field images were taken prior to fluorescent movies to enable identification of cell area, while quantum dot movies enabled the calculation of the force maps. Yellow indicates cardiomyocyte; magenta indicates a fast-migrating cell as detected by deformation of different pillars on different frames. Only cardiomyocyte forces were quantified. (*b*) Zoom into box from (*a*) for detailed force maps during diastole and systole. (*c*) Analysis of diastolic and systolic pillar displacements after 48 h in culture. *n* = 10 and 9 cells for laminin and fibronectin, respectively, from three independent experiments. ***p < 0.001. *p*-values from Student's *t*-test (fibronectin versus laminin).

The different temporal evolution of mechanosensitive behaviour suggested differences in adhesion formation and signalling depending on the ECM ligand. Since adhesion formation and cell morphology are closely related to adhesive forces, we next investigated cardiomyocyte contraction forces that were transmitted through the different integrin/ECM combinations, using nanopillars (figure 2). Movements of pillars coated with either fibronectin or laminin were recorded at frame rates of greater than 10 fps. From the pillar movements, the maximum displacement (systole) was compared to the subsequent minimum (diastole) and to the non-displaced pillars and the contraction force was calculated as the difference between systolic and diastolic force (figure 2*a–c*). Consistent with a difference in spread area and mechanosensing, we found indeed higher forces (both systolic and diastolic) from the NRCs that were plated on the fibronectin-coated pillars.

### (c) Micropatterning indicates enhanced adhesion formation on fibronectin versus laminin

**Figure 3.**
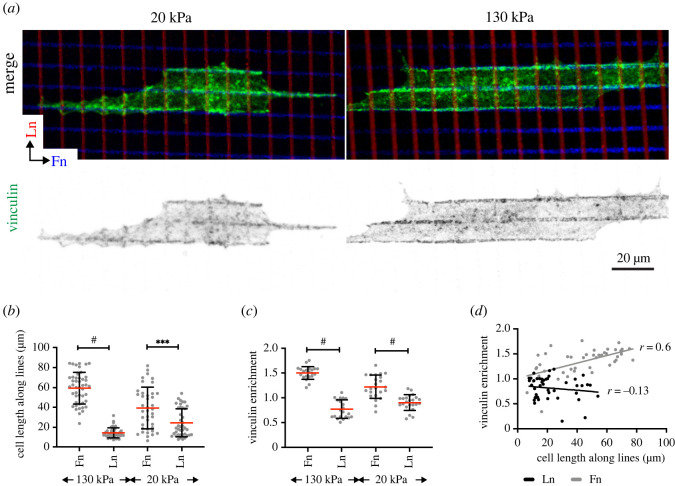
Vinculin enrichment to fibronectin indicates adhesion reinforcement. (*a*) Representative images, (*b*) quantification of cell extent along the respective directions of the grids. *n* = 46 and 40 cells for 130 kPa and 20 kPa conditions, respectively. (*c*) Analysis of vinculin enrichment to the grids and (*d*) Pearson's correlation coefficient of vinculin enrichment versus cell extent. Light grey, fibronectin; dark grey, laminin. *n* = 21 and 23 cells at 130 kPa and 20 kPa conditions. ***, *p*< 0.001; ^#^*p* < 0.0001. *p-*values from Student's *t*-test.

Micropatterning perpendicular lines of different proteins offers an elegant way to analyse the differential interaction of cells with two or more receptor ligands, which we previously employed to study the immune synapse formation in T cells [18]. This method allows direct comparison of the adhesive strength by analysing the relative alignment of the cells with one ligand over the other. Additionally, it allows the measurement of enrichment of adhesion proteins over the ligand lines. Therefore, we decided here to pattern perpendicular lines of fibronectin versus laminin (figure 3). To further investigate the mechanosensing we decided to pattern on PDMS of different stiffness. Confirming the results from the uniformly coated surfaces, we found stronger alignment of the NRCs with the fibronectin lines compared to the laminin lines. This result was more pronounced on 130 kPa versus 20 kPa (figure 3*b*).

Forces on talin reveal cryptic binding sites for vinculin and adhesion reinforcement [19]. Therefore, the respective amount of vinculin can be used as a surrogate measurement of adhesion reinforcement. Indeed, vinculin showed a strong enrichment along the fibronectin lines that was further enhanced at 130 kPa versus 20 kPa (figure 3*c*). Moreover, we found a correlation between vinculin enrichment and spread length along the fibronectin lines, but not between vinculin enrichment and spread length along the laminin lines (figure 3*d*). Together these data indicated that adhesion strength and adhesion forces are more pronounced on fibronectin compared to laminin and result in enhanced vinculin recruitment and adhesion reinforcement.

### (d) DNA origami for controlled presentation of receptor ligands

Since our findings so far demonstrated unique adhesive behaviours of NRCs when cultured on fibronectin compared to laminin, we hypothesized that these might be related to the specific adhesion structure and composition. Indeed, investigations have shown key differences in activation [20] and clustering [15] of different integrin subtypes.

**Figure 4.**
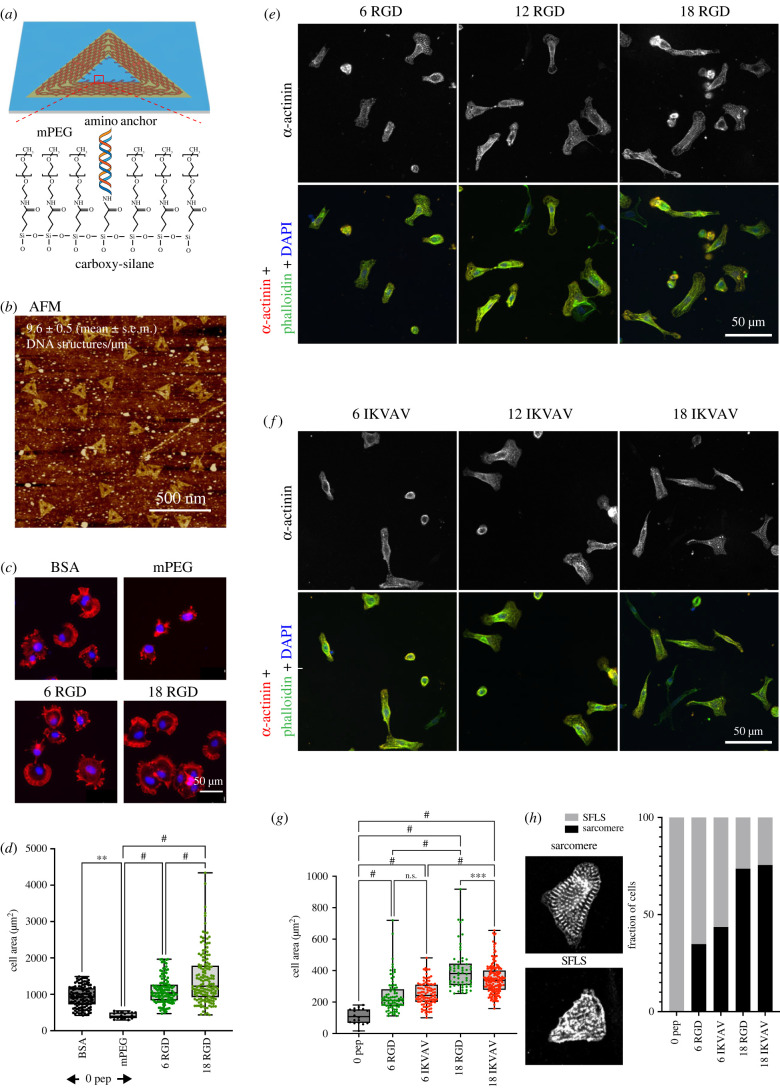
Spreading on random origami scales with integrin–ligand spacing. (*a–d*) Optimization of conditions using mouse embryonic fibroblasts cultured on random DNA origami arrays. (*a*) mPEG-amine was used to block remaining carboxyl groups after attachment of DNA origami via amino anchors to carboxyl-terminated silanes. (*b*) Atomic force microscopy (AFM) image of randomly positioned DNA origami. (*c*) Poly(ethylene glycol) methyl ether (mPEG)-amine blocking prevents non-specific adhesion to the background on DNA origami functionalized with 0, 6 or 18 RGD. Red: phalloidin, blue: DAPI. (*d*) Quantification of *n* = 3 independent experiments. One-way ANOVA with Tukey correction for multiple comparisons, **p < 0.01, ^#^*p* < 0.0001. (*e–h*) NRCs were cultured on RGD or IKVAV functionalized nanopatterns for 24 h. (*e*,*f*) Representative images of NRCs spreading on the RGD (*e*) and IKVAV (*f*). Spreading was quantified by measuring cell area (*g*). (*h*) Maturity was assessed, by counting the number of cells containing stress fibre like structures (SFLS) or additionally contained mature sarcomeres. ^#^*p* < 0.0001. Data pooled from three independent experiments with *N* > 50 cells per condition, except 0 peptide control (*N* = 18; note only few cells attached to this condition). DAPI, 4′,6‐diamidino‐2‐phenylindole.

**Figure 5.**
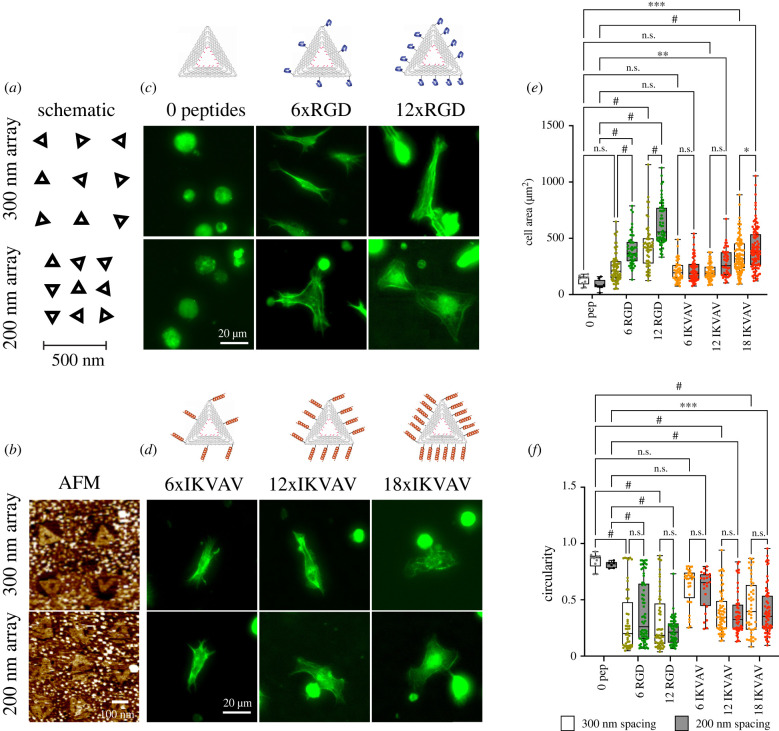
Bionanoarrays for study of nanoscale adhesion organization. (*a*) Schematic of bionanoarrays set-up; since circular patterns are used, distance but not orientation of the origami triangles can be controlled. (*b*) AFM images of DNA origami bionanoarrays. (*c*,*d*) Representative images of NRCs spreading on the RGD (*c*) and IKVAV (*d*) nanopatterns. The schematic on top shows the approximate placing of the peptides. Cells were stained with phalloidin and analysed for cell area (*e*) and circularity (*f*). Differences between peptide numbers and origami spacing were evaluated with a Two-way ANOVA with Tukey corrections for multiple comparisons, **p < 0.01, ***p < 0.001, ^#^*p* < 0.0001. Data from three independent experiments with *N* > 50 cells per condition.

**Figure 6.**
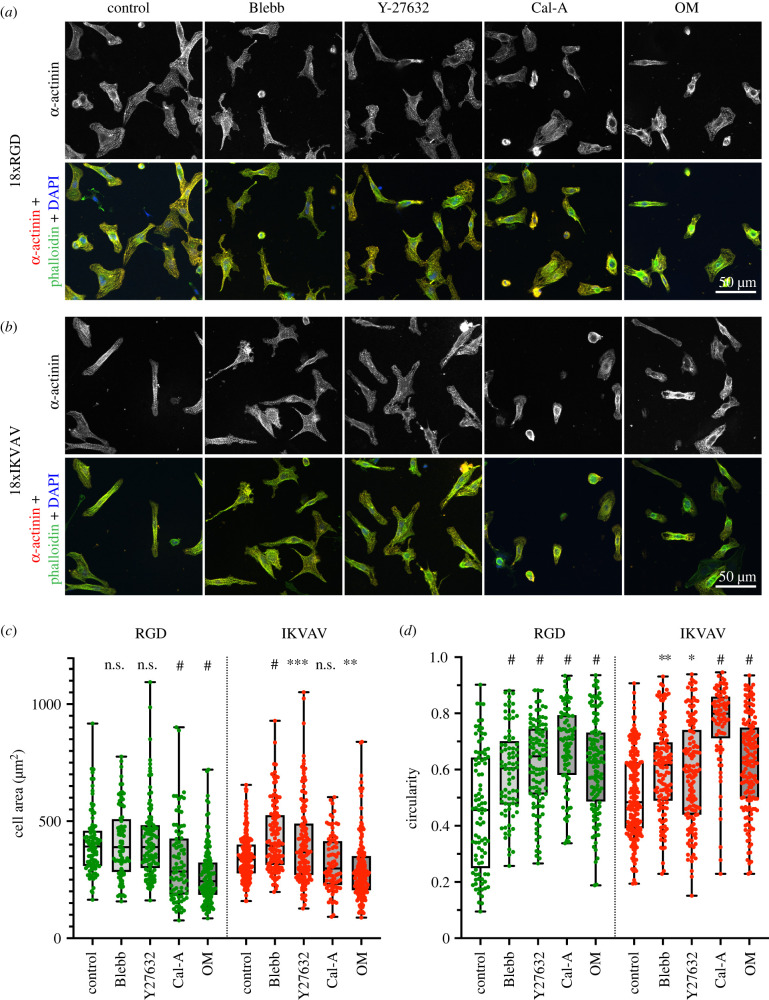
Effect of cytoskeletal contractility inhibitors and activators on NRCs spreading on DNA origami. NRCs were cultured on random origami arrays presenting 18xRGD (*a*) or 18xIKVAV (*b*) peptides and treated with drugs to inhibit (blebbistatin (5 µM), Y-27632 (10 µM)) or promote cytoskeletal contractility (Calyculin-A (100 nM), Omecamtiv Mercabil—OM (500 nM). (*a,b*) Representative images of NRCs immunostained for α-actinin and phalloidin. (*c*) Quantification of cell area. (*d*) Quantification of circularity. One-way ANOVA with Tukey correction for multiple comparison, *p < 0.05, **p < 0.01, ***p < 0.001, ^#^*p* < 0.0001. *N* = 3 independent experiments with *N* > 50 cells per condition.

To understand the role of these factors for the regulation of cardiomyocyte adhesion to the ECM, we decided to employ DNA origami as a tool to control the distance between integrin–ligands with nanometre resolution (figures 4–6). To investigate the nanoscale organization and clustering of fibronectin- and laminin-binding integrins in cardiomyocytes, we used DNA origami presenting 0, 6 or 18 RGD (for fibronectin), or isoleucine–lysine–valine–alanine–valine motif (IKVAV) peptides (to mimic laminin). At a side length of 127 nm per DNA origami triangle, 6 ligands corresponded to an inter-ligand spacing of approximately 60 nm, 12 ligands to approximately 30 nm inter-ligand spacing and 18 ligands were spaced by approximately 20 nm each.

DNA origami were cast on glass substrates at a concentration of 2 nM and cross-linked via amino anchors to carboxyl-terminated silanes of the functionalized glass, leading to a density of 9.6 ± 0.5 DNA structures per µm^2^ (electronic supplementary material, figure S2). Remaining carboxyl groups were subsequently blocked by cross-linking to poly(ethylene glycol) methyl ether (mPEG)-amine and successful passivation was confirmed by plating of mouse embryonic fibroblasts (MEFs, figure 4*a–d*).

Like the MEFs, NRCs were unable to spread on zero peptide controls (with mPEG passivation), confirming that non-specific adhesion was minimal. Upon presentation of six peptides per origami (approx. 60 ligands per μm^2^), NRCs started to spread on both RGD and IKVAV, with no significant differences observed between the two peptides. In general, NRCs were small on 6xRGD and 6xIKVAV arrays (240 ± 107 µm^2^ and 252 ± 76 µm^2^ for RGD and IKVAV, respectively) and most NRCs exhibited primarily stress-fibre like structures (SFLS) with few mature sarcomeres (figure 4*e–h*).

By contrast, the cell area was considerably larger on both RGD and IKVAV arrays presenting 18 ligands (approx. 180 ligands per μm^2^). Here, significantly more cell spreading was observed on RGD compared to IKVAV. Nevertheless, most cells on both 18xRGD and 18xIKVAV were able to form mature sarcomeres. These findings clearly demonstrate that both RGD and IKVAV functionalized DNA origami facilitate NRC spreading and cytoskeletal maturation in a dose-dependent manner, whereby presentation of RGD peptides resulted in larger cell areas compared to IKVAV, but comparable maturity.

### (e) Different nanoscale behaviour of integrins regulates cardiomyocyte adhesion

The investigation of NRCs on randomly positioned DNA origami functionalized with RGD or IKVAV demonstrated that this platform can mediate NRC spreading and adhesion. However, the random arrays can only provide information on integrin clustering relative to the area of a single origami structure (approx. 85 nm^2^), with little control over the global positioning of the origami. Nanopatterned DNA origami (here bionanoarrays) overcomes these limitations by providing precise control of both global and local integrin–ligand placement. Specifically, we generated circular pattern with 150 nm diameter, using e-beam lithography (EBL). After sialinization (using carboxyl-terminated silanes as above) and removal of the remaining resist, DNA origami were conjugated to the carboxyl groups, present only at the specified locations (figure 5*a*, atomic force microscopy (AFM) images).

To compare the global and local minimal ligand densities and clustering behaviours of different cardiomyocyte integrins, bionanoarrays were fabricated with 0 peptides, 6 or 12 RGD or 6, 12 or 18 IKVAV peptides in arrays with inter-origami spacings of 200 nm and 300 nm (figure 5; electronic supplementary material, table S1 summarizes the local and global ligand properties of each nanopattern configuration). Bionanoarrays with zero peptides were employed as negative controls.

Using such bionanoarrays enabled us to specifically study minimal inter-ligand spacings and global densities in order to facilitate cardiomyocyte spreading. Again, on zero peptide controls, few cells adhered to the substrates, suggesting a low level of non-specific adhesion. The addition of 6xRGD peptides (approx. 60 nm spacing) resulted in an increase in cell attachment and cell area and a decrease in circularity (figure 4). This indicated that the local ligand density was sufficient to allow at least some level of spreading (similar to previous work in migratory cells) [21].

Nevertheless, on arrays with 200 nm cluster spacing (6xRGD/200 nm; 150 peptides µm^–^^2^), the cardiomyocyte area was significantly larger than on 6xRGD/300 nm bionanoarrays (69 peptides µm^–^^2^). Increasing the number of RGD peptides to 12 (decreasing inter-ligand spacing to approx. 30 nm) resulted in a further increase in cell area on both, 12xRGD/200 nm and 12xRGD/300 nm arrays. Interestingly, NRC area on the 12xRGD/300 nm bionanoarray was comparable to the 6xRGD/200 nm array. Since both arrays had comparable overall ligand densities (electronic supplementary material, table S1, 139 peptides µm^−2^ versus 150 peptides µm^−2^), this result clearly suggested a dominance of global over local ligand density for fibronectin-binding integrins in cardiomyocytes.

On IKVAV bionanoarrays in contrast, only few cells were spread on either 6xIKVAV/200 nm or 6xIKVAV/300 nm arrays, suggesting insufficient integrin clustering and adhesion formation. This was also apparent from the cell morphology with higher circularity indicating overall lesser amount of spreading and maturation compared to the corresponding RGD arrays (figure 5*d*). With 12xIKVAV peptides, a significant cell area increase was only observed on 12xIKVAV/200 nm arrays, although the change in cell shape was testament to an activation of integrin signalling and adhesion stabilization also on 12xIKVAV/300 nm arrays (figure 5*c*,*d*). Together, this suggested that cardiomyocyte laminin-binding integrins required a smaller inter-ligand distance of approximately 30 nm versus approximately 60 nm to enable integrin adhesion formation and spreading.

Further spreading was facilitated by adding 18xIKVAV ligands per DNA origami (approx. 20 nm inter-ligand spacing). Here, cardiomyocytes again displayed larger cell areas on 18xIKVAV/200 nm arrays compared to 18xIKVAV/300 nm arrays. However, spread area was still significantly less than on 12xRGD/200 nm arrays (and comparable to 12xRGD/300 nm arrays). Importantly, cardiomyocyte area was larger on 18xIKVAV/300 nm compared to 12xIKVAV/200 nm bionanoarrays, even though the former had a lower global ligand density of 208 versus 300 peptides µm^−2^. This was in contrast to RGD bionanoarrays and suggested a reduced role of global compared to local densities for laminin- versus fibronectin-binding integrins in neonatal cardiomyocytes.

In summary the bionanoarrays indicated different minimal inter-ligand spacings (30 nm versus 60 nm for IKVAV and RGD, respectively) and different contributions of local and global ligand densities that are needed for activating integrin signalling and supporting stable cell adhesion and spreading.

### (f) The integrin ligand affinity is balanced with the cytoskeletal contractility

Since we found differences in spread area, cytoskeletal arrangements and adhesion signalling, we next thought to investigate the source of this discrepancy. Previously we found a role for myofibrillar and non-myofibrillar tension in regulating cardiomyocyte adhesion formation and mechanosensing [5]. To investigate this further, NRCs spreading on random 18xRGD or 18xIKVAV arrays were treated with the contractility inhibitors blebbistatin (to inhibit myosin II) and Y-27632 (to inhibit ROCK) or contractility enhancers Calyculin-A (inhibits myosin-light-chain phosphatase from dephosphorylating myosin [22]) and Omecamtiv Mercabil (OM, binds to the catalytic domain of myosin and increases cardiac contractility [23]) (figure 6).

Reducing contractility with blebbistatin or Y-27632 resulted in a significant increase in cell area on IKVAV (but not on RGD). This result suggests that the adhesions to IKVAV at the specific ligand concentrations employed here are unstable at the existing levels of contractility. Therefore, reducing the contractility stabilized adhesions and facilitated spreading. By contrast, drugs promoting contractility resulted in a decrease in cell area on both RGD and on IKVAV functionalized DNA origami nanoarrays. The decrease was stronger with OM and only this treatment showed a significant effect on IKVAV modified surfaces.

**Figure 7.**
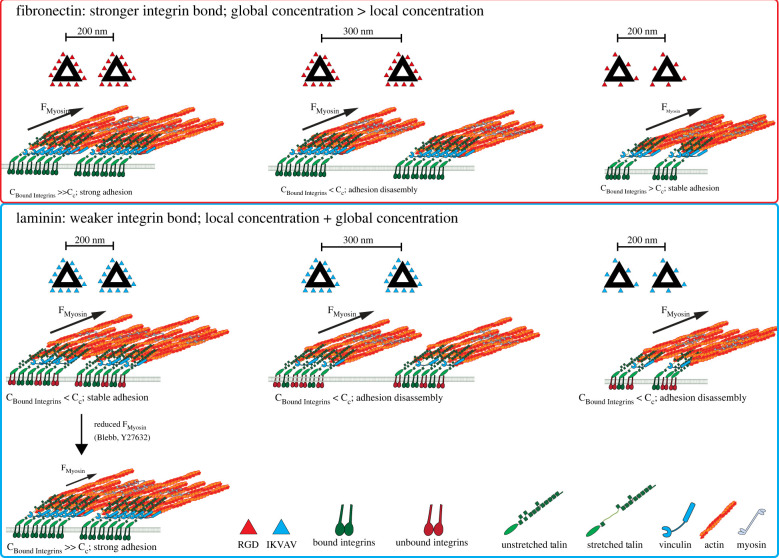
Model for nanoscale requirements of fibronectin- and laminin-binding integrins in cardiomyocytes. Top panel: fibronectin-binding integrins establish relatively strong bonds to their ECM ligands and/or talin and cytoskeletal forces lead to talin stretching, vinculin binding and adhesion reinforcement (i.e. the concentration of bound integrins, C_Bound Integrins_, is larger than the required critical concentration, C_c_). Stable adhesion clusters can be formed with a smaller number of ligands and because of the higher stability of bonds, larger distances between clusters can be sustained. Overall, the global concentration is more important for determining the adhesion stability than the local concentration. Bottom panel: weaker bonds of laminin-binding integrins either to the ECM ligand and/or talin result in more frequent bond ruptures. This results in an overall lower number of force-bearing integrin–talin links at a certain time (i.e. the concentration of bound integrins, C_Bound Integrins_, is smaller than the required critical concentration, C_c_). A larger number of ligands is required to stabilize the adhesion clusters. Hence local, as well as global concentrations of integrins determine the adhesion strength. Reducing the myosin forces through treatment with blebbistatin or Y27632 increases the bond lifetimes and adhesion stability.

Together, these results suggest that cytoskeletal contractility, and integrin ligand affinity, need to be well balanced to support cell spreading. IKVAV/laminin-binding integrins overall require higher density of ligands to support adhesion formation and downstream signalling and hence cannot support the contractility of neonatal cardiomyocytes to the same extent as *α*5*β*1 integrin adhesions to RGD (see model in figure 7).

## 3. Discussion

During development and in disease, cardiomyocytes express fibronectin-binding integrins (especially *α*5*β*1), while laminin-binding integrins are the dominant subtypes in healthy adult cardiomyocytes [1,10,12]. Previous work demonstrated that different integrin subtypes have unique ligand-binding and rigidity-sensing properties, but the consequences of the distinct integrin signalling on the cardiomyocyte phenotype are not well understood [1,14–16,24,25]. The current investigation made use of an interdisciplinary approach to conduct a comparative analysis of NRC spreading and integrin clustering on fibronectin and laminin functionalized substrates. We demonstrated that cardiomyocytes cultured on fibronectin exhibit stronger spreading, contractility and vinculin enrichment compared to cells cultured on laminin. We hypothesized that these differences could arise owing to differential integrin clustering. Indeed, by implementing a single molecule ligand nanopatterning platform, we find that cardiomyocytes spreading on laminin-binding integrins require more concentrated integrin clusters to initiate spreading, compared to RGD-binding integrins. Our data further indicate differences in the requirements for local (nanometre-scale) versus global (micrometre-scale) ligand densities and that these differences are driven by the respective stability of the adhesions to existing levels of cytoskeletal contractility.

While the functional implications of differential integrin expression in cardiomyocytes are poorly understood, we can look to other cell types that modulate integrin expression profiles to perform specific functions (e.g. migration, adhesion formation). The different integrins possess unique ECM-bond dynamics (e.g. binding/unbinding rates and catch/slip bond behaviour), which are highly sensitive to force (i.e. contractility and substrate rigidity) and are further characterized by their nanoscale organization and clustering properties [15,26]. Especially, *α*v*β*3-expressing cells required an inter-ligand distance between individual RGD molecules of 60 nm or less, disregarding the global concentration, in order to form stable adhesions [15,27–31]. Moreover, a study employing peptidomimetic ligands specific for *α*v*β*3 and *α*5*β*1 integrins found similar spreading for both integrin types with 60 nm inter-ligand spacing but detected further spreading of *α*5*β*1 integrins at even smaller ligand spacings [15].

Little was known about the clustering behaviours of fibronectin and laminin integrin subtypes in cardiomyocytes, especially *α*5*β*1 and *α*7*β*1 [32,33]. Here, we observed the same 60 nm threshold for RGD-binding integrins. However, unlike observations in fibroblasts with larger levels of *α*v*β*3 integrins and differences in *β*1 integrin and talin isoforms [21], we found a strong contribution of global in addition to local ligand densities and in fact cardiomyocytes were spread out to the same extents on similar global concentrations of RGD peptides, independent of the spacing of the ligands on each cluster or the distance between the clusters. Intriguingly, for cardiomyocytes, laminin-binding integrins required much smaller spacings of approximately 30 nm (or 12 peptides per cluster) to initiate spreading, compared to the fibronectin-binding integrins. Moreover, there seemed to be a stricter requirement for the local, in addition to global ligand concentrations. This suggested a difference in the ability to form adhesions over multiple clusters and stably distribute the adhesion forces over larger distances between the clusters (see model in figure 7). In other words, for RGD binding integrins, a 200 nm inter-cluster spacing can be bridged with only 6 ligands per cluster; however, at least 12 ligands per cluster are required in order to bridge the 300 nm distance. A stronger requirement for inter-ligand versus inter-cluster spacing for IKVAV binding integrins, in turn, agrees with an overall lower adhesion force and stability and hence an inability to effectively bridge clusters even at 200 nm distance.

Integrin clustering is a critical part of adhesion formation and responds to molecular tension. Integrins demonstrate typical catch bond behaviour and extend the bond lifetimes to their ligand under the application of force [6]. Additionally, the integrin-binding protein talin is stretched in a force-dependent way, leading to the opening of cryptic vinculin binding sites [19]. Vinculin then provides additional links between talin and actin to reinforce the adhesion [6]. However, if the forces or loading rates are too high, then bonds will break before the adhesion can form. Integrin clustering directly modulates the forces experienced by components of the integrin adhesion; a greater number of integrins enables distribution of traction forces among more proteins, resulting in less force being sensed by individual integrins and adaptors [26].

Pharmacological inhibition of cytoskeletal contractility resulted in an increase in NRC spreading on IKVAV, further suggesting that laminin-binding integrin adhesions (at current ligand densities) may be less stable under high forces. This would also agree with observations of decreased cytoskeletal content, traction forces and spreading in cells cultured on laminin.

Together, these results suggest that fibronectin adhesions mediate stronger spreading, contractility and integrin adaptor enrichment and enhanced stability of individual adhesions. Furthermore, this finding suggests that a transition in integrin expression to favour fibronectin-binding subtypes (especially *α*5*β*1) may also alter integrin signalling and mechanotransduction pathways [34,35].

To specifically analyse the integrin–ligand interactions, we used here micro- and nanopatterned two-dimensional surfaces. The use of two-dimensional surfaces comes with the limitation that upon plating, cardiomyocytes remodel to form peripheral focal adhesion structures in addition to costameric structures at the level of the z-disc (e.g. visible by a striated vinculin staining) [5]. Nevertheless, the presence of fibronectin- and laminin-binding integrins makes NRCs an excellent model to study the nanoscale adhesion properties. However, future work will need to analyse the contributions of the different *β*1 integrin and talin isoforms, which differ in their affinities to each other [16].

In summary, the current results provide novel insights into the cardiomyocyte integrin adhesions during postnatal heart development or in heart disease, where we find that the switch in cardiomyocyte integrin expression has important implications for integrin signalling and mechanotransduction.

## 4. Methods

### (a) Cells

NRCs were prepared as described previously [5]. Hearts were dissected into ice-cold ADS buffer (116 mM NaCl, 20 mM Hepes, 0.8 mM NaH_2_PO_4_, 5.6 mM glucose, 5.4 mM KCL, 0.8 mM MgSO_4_). After hearts settled down to the bottom, they were washed once with ADS buffer. ADS buffer was then removed, and hearts were incubated with 5 ml enzyme solution in ADS (ES, 246U collagenase and 0.6 mg pancreatin ml^−1^), for 5 min, at 37°C under vigorous shaking. Supernatant was discarded. This step was followed by five to six digests, until hearts were completely digested. Each time, 5 ml fresh enzyme solution (ES) was added to the hearts and incubated 15 min at 37°C, under shaking. Hearts were pipetted up and down 30 times using a pasteur pipette. After settling down, supernatant was transferred into plating medium (65% DMEM, 17% M199, 10% horse serum, 5% fetal bovine serum (FBS), 2% GlutaMAX, 1% penecillin/streptamycin (P/S)). Two digests each were combined in one tube with 20 ml plating medium, then cleared through a 100 µm cell strainer and spun down at 1200 r.p.m. for 5 min at RT, before being resuspended in 10 ml plating medium. Cells were pooled together and pre-plated for 90 min to enrich the cardiomyocytes. Cardiomyocytes were then plated onto the respective substrates as indicated in the text or figures. Medium was changed the next day to maintenance medium (77% DMEM, 18% M199, 2% horse serum, 2% GlutaMAX, 1% P/S), or serum starvation medium (as above, but excluding the horse serum).

The following drugs were applied for 2 h in serum-free medium: Y27632 (10 µM; Tocris); blebbistatin (5 µM; Calbiochem); Calyculin-A (100 nM; Sigma). Adult rat cardiomyocytes were isolated by Langendorff perfusion of hearts as described previously [17]. Adult and newborn rats were sacrificed in accordance with the Schedule 1 to the Animals (Scientific Procedures) Act 1986.

### (b) Immunostainings

For immunostaining, cells were fixed with 4% paraformaldehyde (PFA) for 10 min, permeabilized with 0.2% triton X-100 in PBS for 5 min, blocked with 5% BSA in PBS for 1 h and stained in the antibody solutions in immunostaining buffer (20 mM Tris, 155 mM NaCl, 2 mM EGTA, 2 mM MgCl_2_, 1% BSA at pH 7.4) [17]. Cells were washed three times for 10 min with PBS after each step and mounted in MOWIOL 4–88 (0.1 g ml^−1^ in Glycerol/Water/Tris (0.2 M, pH8.0) at a ratio of 1/1/2) containing a final concentration of 4% n-propyl gallate. Live cell imaging and imaging of multiwell plates were performed on an inverted Nikon Eclipse Ti-E microscope with a Nikon DS-Qi2 sCMOS camera and equipped with a Solent Scientific chamber with temperature and CO_2_ control. Confocal microscopy was performed on a Nikon A1R+ inverted microscope with GaAsP Detectors. TIRF imaging and bleaching curves were recorded on a Zeiss LSM710 Elyra Microscope.

### (c) Polydimethylsiloxane substrates and nanopillars

PDMS pillar (500 nm diameter, 1.7 µm height, 1 µm centre-to-centre) substrates were prepared by soft lithography from silicon masters as described previously [36]. For fluorescent labelled pillars, CdSeS/ZnS alloyed quantum dots (490 nm, Sigma) were spun first on the master 30 s at 10 000 r.p.m. with a 150i spin processor (SPS), before the addition of PDMS. PDMS (Sylgard 184, Dow Corning) was mixed thoroughly with its curing agent (10 : 1), degassed, poured over the silicon master, placed upside-down on a plasma-treated coverslip-dish (Mattek) or coverslip four-well dishes (Ibidi) and cured at 80°C for 2 h. The mould was then removed and the pillars were incubated with fibronectin for 1 h at 37°C.

Flat PDMS substrates were prepared by spin coating Sylgard 184, Sylard 527 or mixtures at the Ratios of 1 : 5, 1 : 10 and 1 : 20 with a 150i SPS, onto coverslips for western blotting samples, or microscope slides for placing into multiwell plates (Grace Biolabs). Before spin coating, Sylgard 527 was pre-cured at 70°C for 30 min with intermittent mixing to achieve a comparable viscosity to the Sylgard 184 mixture.

### (d) Micropatterning

Microcontact printing was performed as described elsewhere [18]. Briefly, hPDMS stamps were cast on E-beam-lithographed poly(methyl methacrylate) (PMMA) wafers. 20 µg ml^−1^ ligand proteins in PBS were deposited onto the stamps for 40 min. Micro-patterns were printed by stamping on plasma-treated glass for 1 min. Fibronectin/laminin micro-grids were printed in two steps: (i) fluorescent fibronectin deposition followed by (ii) printing of transverse fluorescent laminin lines. Cells were seeded after washing and blocking with 5%BSA.

### (e) DNA origami

DNA origami was assembled as described previously [37]. Briefly, the 7249 base single-stranded circular M13mp18 bacteria phage DNA (5 nM) and staple strands (50 nM) were combined in 50 µl of TAE (Tris-acetate-EDTA) buffer with 12.5 mM Mg^2+^ (DNA sequences can be obtained at [38]). An appropriate quantity of ions, such as magnesium here, or sodium, are required for efficient DNA hybridization. This acts to equilibrate electrostatic repulsion between highly negatively charged DNA molecules. An amount of 12.5 mM Mg^2+^ was found to be sufficient to achieve a high yield of DNA origami and limit any aggregation effects. DNA origami are synthesized by annealing from an initial temperature of 94°C to completely melt all dsDNA. Temperature step-controlled annealing was carried out in a PCR machine. Samples were cooled from 94°C to 65°C at a rate of approximately 0.3°C per minute. A cooling rate of 0.1°C per minute is employed from 65°C to room temperature. The self-assembled DNA origami were then purified using Millipore Amicon Ultra 100 kDa spin columns in a centrifuge at 2000 rcf for 6 min, three times, to remove excess staple strands. DNA origami were adjusted to a concentration of approximately 20 nM and stored in Lo-Bind Eppendorf tubes at 4°C. A NanoDrop spectrophotometer is used to detect the approximate concentration of DNA origami products, assuming a molecular weight of 330 g mol^−1^ per base and an extinction coefficient (260 nm) of 33 mg ml^−1^ for the calculation. To ensure efficient assembly and labelling of DNA origami with peptide conjugates, unmodified staple strands and amino anchors were added at a 5× excess to the M13mp18 backbone and peptide conjugates were added at a 10× excess.

RGD (cyclo(Arg‐Gly‐Asp‐D‐Phe‐Cys) and IKVAV (laminin AChain, 2091‐2108, Cys‐Ser‐Arg‐Ala‐Arg‐Lys‐Gln‐Ala‐Ala‐Ser‐Ile‐Lys‐Val‐Ala‐Val‐Ser‐Ala‐Asp‐Arg) peptides were conjugated to ssDNA through UV-mediated thiol-ene reaction, via a sulfhydryl group on the peptides and a thiol group on acrydite modified ssDNA (Integrated DNA Technologies). Peptides were diluted to 100 µM in H_2_O containing 100× TCEP pH 7.0, to reduce any disulfide bonds and present the cysteine groups for conjugation. Peptides were then mixed with the acrydite-modified ssDNA at a final concentration of 20 µM and 200 µM, respectively. Reactions were carried out in 120 mM Tris buffer with 11 µM photoinitiator (2-hydroxy-4′-(2-hydroxyethoxy)-2-methylpropiophenone). Samples were exposed to 260 nm UV light for 1 h and the conjugates were purified by RP-HPLC and freeze drying, as described above.

DNA origami were characterized by atomic force microscopy (AFM) (Bruker, Dimension Icon) as described previously [37].

### (f) Peptide and amino modifications to DNA origami

The pointed triangle design was selected owing to its lower tendency to aggregate and to exhibit blunt end stacking effects [39]. Eighteen peptide and 15 amino anchor modifications were made to the triangle origami design. For the peptide modifications, the original strands that hybridize to the M13mp18 backbone were extended with the same sequence (AGTTGTGGATCCTACT). This extended sequence was complementary to the acrydite peptide linker when it was conjugated to the peptide. Strands selected for the amino anchors were modified with a poly-T extension, complementary for poly-A 6 carbon amino anchor. All modifications were reported previously [37]. During synthesis of the DNA origami, peptide and amino modification were added at a 20× excess to the concentration of the available modification sites.

### (g) Nanopatterning

Nanopatterns were produced by direct write EBL on coverslips. Coverslips were solvent cleaned in an ultrasonic bath (acetone followed by methanol, IPA and water) and dehydrated in a 180°C oven overnight. An oxygen plasma treatment for 60 s at 100 W power prepared the surface for silane deposition, which was done from hexamethyldisilazane (HMDS) vapour in a closed container at 150°C. A 950 000 g mol^−1^ PMMA film was spin coated on the coverslips at 5000 r.p.m., followed by a short solvent bake on a 180°C hotplate for 30 s. A 10 nm aluminium charge conduction layer was evaporated on the coverslips, and they were mounted on a four-inch silicon wafer for EBL processing using crystalbond 555 adhesive. EBL exposure was carried out as described previously [40] to define arrays of 200 nm and 300 nm pitch holes, diameter 150 nm, in the PMMA layer. After exposure, the aluminium charge conduction layer was removed in a 2.6% tetramethylammonium hydroxide (TMAH) solution (MF-CD26) for 30 s followed by water rinse.

For patterning of DNA origami on e-beam patterned substrates, patterned substrates were first developed in a 2 : 1 solution of propanol-2 : methyl isobutyl ketone for 60 s at 23°C, followed by a 30 s wash in 100% propanol-2. DNA origami binding sites were then etched using oxygen plasma treatment (100 W, 70 s, room air) followed by silanization with 0.1% 3-chloropropyltriethoxysilane (CTES) in Tris buffer (5 mM, pH 8.0). The PMMA resist was then removed by immersion in *N*-methyl-2-pyrrolidone (NMP) at 50°C and sonicated or 10 min. DNA origami were incubated for 1 h at room temperature at a concentration of 1 nM in Tris buffer (5 mM, pH 8.3, 50 mM MgCl_2_; see supplementary methods for more information on placement conditions). Owing to the hydrophobic nature of the HMDS layer and the size of the patterned area, 100 µl of origami solution were required to cover the surface sufficiently. Samples were then washed with 3-(N-morpholino)propanesulfonic acid (MOPS) buffer (10 mM, pH 8.1, 50 mM MgCl_2_) to remove primary amines, followed by a wash with MOPS buffer (10 mM, pH 8.1, 50 mM MgCl_2_) containing 50 mM 1‐ethyl‐3‐(3‐dimethylaminopropyl)carbodiimide (EDC) and 25 mM sulfo-N-hydroxysulfosuccinimide (sulfo-NHS). Finally, samples were then washed with the MOPS buffer without MgCl_2_ and rinsed with Dulbecco's phosphate-buffered saline (DPBS) containing 125 mM NaCl. Before AFM characterization, samples were placed into deionized H_2_O.

### (h) Image analysis

Pillar displacements were analysed with imageJ, using the NanoTracking plugin. An image of the pillars after removal of the cells with 10× trypsin was taken as reference for the non-displaced pillars. For contraction analysis, pillar displacements from spontaneous contracting cardiomyocytes were measured for the whole movie, using Matlab. From the data, the maximum displacement (systole) was compared to the subsequent minimum and to the non-displaced pillars. Noise levels were measured from pillars outside the cell and estimated around 30 nm (consistent with our previous work) [5]. Therefore, all pillars that were displaced above 30 nm during the movie were considered for the analysis. Statistics were calculated between cells. Cell area, cell morphology and staining intensity were analysed with cell profiler.

### (i) Quantification and statistical analysis

Datasets were tested for normal distribution using the Shapiro–Wilk test. All box plots are displayed as median (central line), upper and lower quartile (box), ±1.5× inter quartile range (whiskers). All *n*-numbers and statistical tests are indicated in the figure legends. All statistical tests were performed with Graphpad Prism.

## Data Availability

All data needed to evaluate the conclusions in the paper are present in the paper and/or the electronic supplementary material [41].
